# Ancient Leishmaniasis in a Highland Desert of Northern Chile

**DOI:** 10.1371/journal.pone.0006983

**Published:** 2009-09-10

**Authors:** Maria Antonietta Costa, Carney Matheson, Lucia Iachetta, Agustín Llagostera, Otto Appenzeller

**Affiliations:** 1 Instituto Investigaciones Arqueológicas y Museo, Universidad Católica del Norte, San Pedro de Atacama, Chile; 2 Paleo-DNA Research, Lakehead University, Thunder Bay, Ontario, Canada; 3 Department of Anthropology, The University of Western Ontario, London, Ontario, Canada; 4 New Mexico Health Enhancement and Marathon Clinics Research Foundation, Albuquerque, New Mexico, United States of America; University of Oxford, United Kingdom

## Abstract

**Background:**

Leishmaniasis is an infectious disease endemic today in many areas of South America.

**Methodology:**

We discovered morphologic and molecular evidence of ancient infections in 4 female skulls in the archaeological cemetery of Coyo Oriente, in the desert of San Pedro de Atacama, Northern Chile. The boney facial lesions visible in the skulls could have been caused by a number of chronic infections including chronic Leishmaniasis. This diagnosis was confirmed using PCR-sequenced analyses of bone fragments from the skulls of the affected individuals.Leishmaniasis is not normally found in the high-altitude desert of Northern Chile; where the harsh climate does not allow the parasite to complete its life cycle. The presence of Leishmaniasis in ancient skulls from the region implies infection by the protozoan in an endemic area–likely, in our subjects, to have been the lowlands of North-Eastern Argentina or in Southern Bolivia.

**Conclusions:**

We propose that the presence of the disease in ancient times in the high altitude desert of San Pedro de Atacama is the result of an exogamic system of patrilocal marriages, where women from different cultures followed their husbands to their ancestral homes, allowing immigrant women, infected early in life, to be incorporated in the Atacama desert society before they became disfigured by the disease. The present globalization of goods and services and the extraordinary facile movement of people across borders and continents have lead to a resurgence of infectious diseases and re-emergence of infections such as Leishmaniasis. We show here that such factors were already present millennia ago, shaping demographic trends and the epidemiology of infections just as they do today.

## Introduction

### Study site

**Figure 1:**
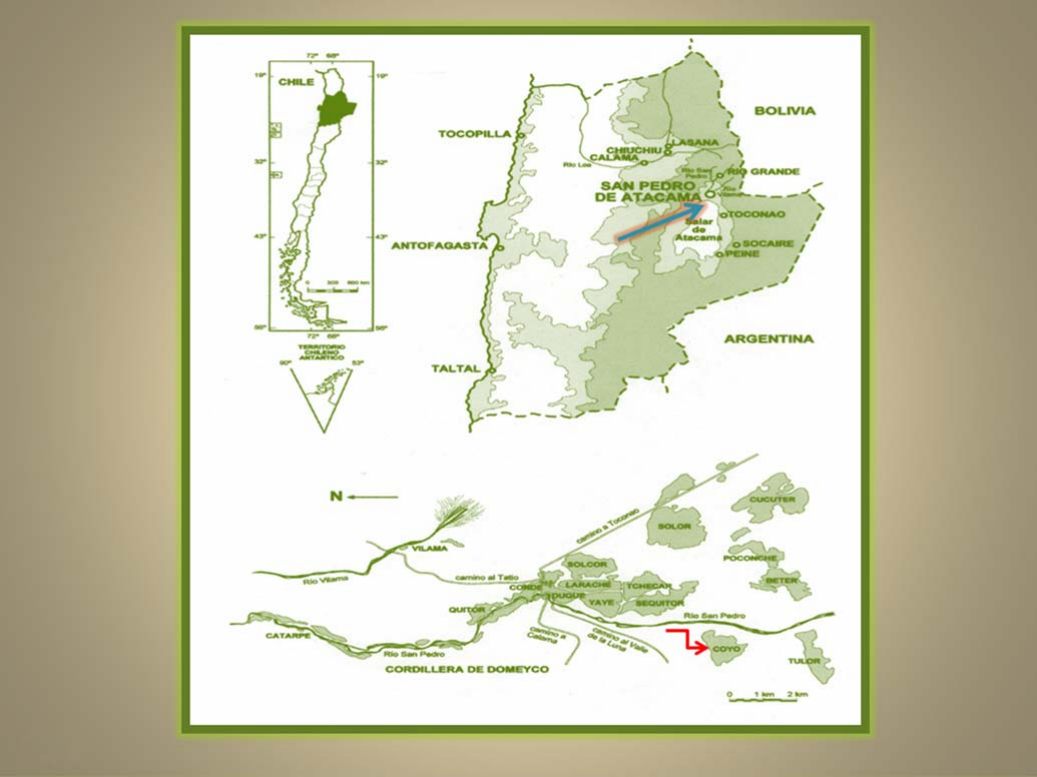
Map of Chile (left) with the district of San Pedro de Atacama indicated in black-fil. Enlarged on the right. The village of San Pedro de Atacama; blue arrow. The archeological sites (digs) are enlarged at the bottom. Cojo Oriental indicated by broken red arrow.

San Pedro de Atacama is situated at 2400 m. altitude in the region of Antofagasta in Northern Chile. This village is located on the Northern slopes of the Salar de Atacama, in the foot-hills of the Andes (Figure 1). Abundant archaeological evidence shows that the area has been inhabited for at least the last 11,000 years.

The Atacama Desert is part of a large arid belt located in western South-America extending from Southern Peru to Northern Chile. The village of San Pedro de Atacama lies in the most arid area, near the Tropic of Capricorn, at one of the many oases that allow limited farming and permanent settlements.

The general aridity has preserved numerous archeological remains in cemeteries from pre-Hispanic times. These archeological specimens are now housed and studied at the Museo Arqueológico of the Instituto de Investigaciones Arqueológicas y Museo of the Universidad del Norte in San Pedro de Atacama.

Our discovery of human skeletal evidence of Leishmaniasis in one of the ancient cemeteries of this arid area was unexpected. Leishmaniasis, common in lower parts of South America, is unlikely to have been endemic in the oases of San Pedro de Atacama in antiquity. A more likely explanation for its pre-historic presence in this arid high altitude desert is that infection occurred in an endemic neighborhood from which the victims migrated to San Pedro de Atacama.

### Leishmaniasis

Leishmaniasis is a polymorphic disease caused by more than 30 species of protozoans of the genus *Leishmania*; there are numerous reservoirs in a variety of mammals. The disease is transmitted through skin puncture by the female of several species of sand flies of the type *Phlebotomus* in the Old World and of *Lutzomyia* in the Americas.

There are three main clinical forms; visceral, cutaneous and mucocutaneous [1].

1. *Visceral Leishmaniasis*, also called Kala-Azar or Dumdum Fever, is found in more than 60 countries worldwide; this is the most serious form, uniformly fatal if untreated. Caused by *L. donovani*, it affects the liver, spleen, lymphatics and bone marrow.
2. *Mucocutaneous Leishmaniasis*. This form is commonest, causing skin lesions that resolve spontaneously within a few months but may recur, if untreated, to form chronic ulcers and cause severe disfigurement around the nose, eyes, and mouth. This variety is also known as *L. selvática* or Espundia and is considered native to South America, where it has been present since antiquity, as evidenced by “huacos,” pottery vases often shaped like human heads that may have had ceremonial, religious, artistic or aesthetic uses in central Andean pre-Columbian civilizations. Many huacos have been found with facial deformations suggestive of chronic Leishmania infection.
3. *Cutaneous Leishmaniasis*. This variety causes skin lesions which resolve spontaneously in a few months. The organism responsible is *L. major*, which is found world wide.

The distribution of human Leishmaniasis is co-extensive with the prevalence of the sand fly vectors and thus constrained by climate, geography and altitude (Figure 1). The climate at the altitude of San Pedro de Atacama (2400 m.) restricts the endemicity of the disease, by impeding the completion of the life cycle of the organism. The nearest region of endemic disease is in the province of Yungas in Northern Argentina (Figure1).

### Socio-cultural aspects

For about 1000 years before the Christian era villages in the Atacama Desert, [2] maintained close ties with the people from Southern Bolivia and North-Western Argentina.The “Atacameños” also exchanged goods and services in what has been called the “Reticular System of Puneño Interaction” [3].

A key trade item sought by the people of the Atacama Desert was the psychotropic seed cebil (*Colubrina Anadenanthera*), found in Argentine in the Yungas, which was used for shamanistic rituals [4].

Previous work on prehistoric paleopathological material from Peru and Chile (from the town of Arica) failed to describe any osseous lesions that could be attributed to mucocutaneous Leishmaniasis [5], [6], [7], [8], [9], [10], [11], [12], [13], [14]. Conversely, a study of skeletal remains, using X-rays and axial tomography, from Lima Peru, a humid, sub-tropical area at sea level, revealed lesions compatible with mucocutaneous Leishmaniasis in 2.07% of the examined bones [15].

The cemetery at Coyo Oriental (Fig. 1) where the disfigured skulls were found dates to between 500 and 1,000 years ago, [16] a period of increasing contacts between Atacameños and the people of the Yungas in Argentina. With the commerce came an increasing flow of foreigners into the Atacama Desert [17], leading to intermarriage and kinship bonds between high altitude desert people and lowlanders from tropical areas.

## Results

We determined the sex and approximate age of the 4 affected skulls using the usual archeological criteria [12], [18], [19] (also see supporting on line information; text S1). No pelvic measurements were available for sex determination. The contents of the tombs where the lesioned skulls were discovered did not differ significantly from those of the other tombs excavated in this cemetery, suggesting that the dead were local people. No other skulls with deformities were found in this region.

**Figure 2:**
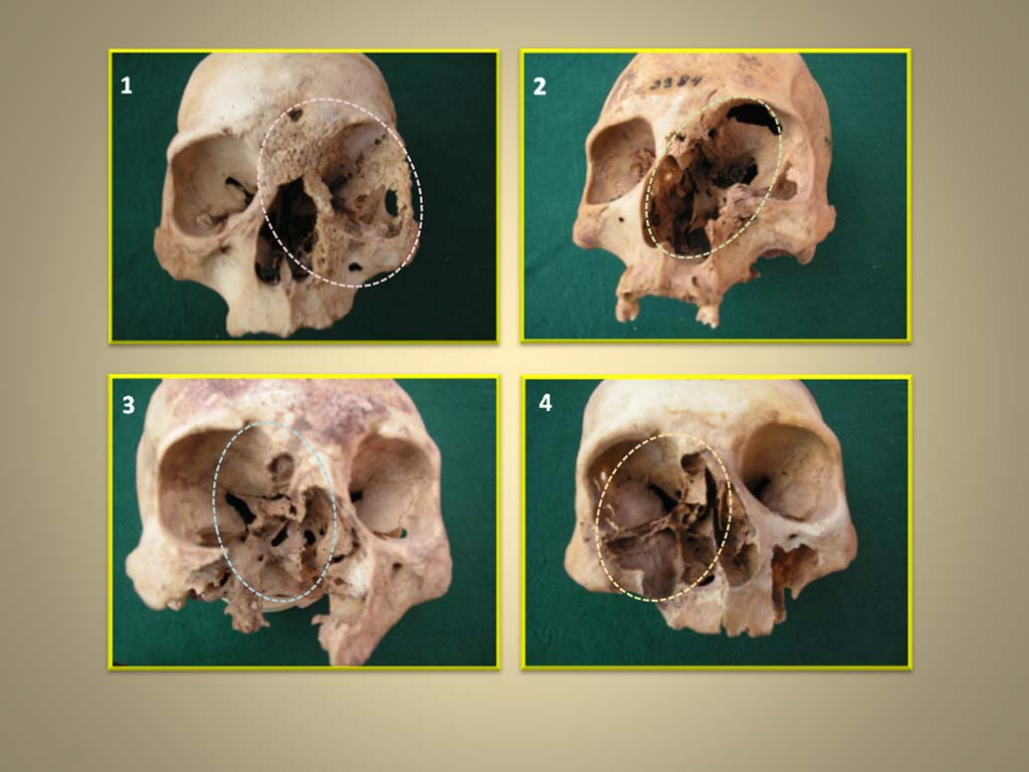
Four female skulls from ancient graves, from Cojo Oriental cemetery, San Pedro de Atcama Northern Chile. They show extensive, destructive lesions (outlined by broken lines) caused by chronic Leishmania infection. 1. Skull # 4156 (12204), age 45–49 years showing facial boney erosions on the left side, with evidence of healing. The grave contained baskets and bags filled with quinoa, wool and human hair. The lesions were extensive involving the orbit, the nasal bones, the maxillary, the ethmoid and sphenoid sinuses, the maxilla and the frontal bone. The inferior orbital rim was destroyed. In the remaining maxilla 2 suppuration tracks were visible. Evidence for periostitis was found in the frontal bone. The hard palate was not involved. The mandible was not examined. 2. Skull # 3984 (9791) aged 30–40 years. Left sided, lesions involving the nose and orbit with evidence of partial healing. The grave contained 3 additioal adults, and also pottery, baskets and 1 pumpkin. Extensive lesions involving the orbit, the nasal bones, the maxillary, the ethmoid and sphenoid sinuses, the maxilla and the frontal bone. There was thinning of the entire orbital circumference. Suppuration tracks were visible in the ethmoid and in the frontal bone. A vertical fracture with depression and displacement towards the left was found between the glabella and the frontal bone. The mandible was normal. 3. Skull # 5377 (9858), age 35–39. Right-sided boney erosions with evidence of healing. The maxilla and nasal bones were missing. The hard palate on the left was also involved. The grave contained 3 additional adults and a large number of ceramics, baskets, a malachite necklace and 1 bag filled with red earth and snail shells, 1 spoon and 3 pumpkins. Deformed porous bone was evident in the region of the right pyramidal process. The zygomatic arch was also deformed by porous boney outgrowth and largely remodeled. Partial destruction of the left hard palate was present. The malar and frontal portions of the orbital rim were thinned but still discernable. The mandible was not involved. 4. Skull # 3938 (14673), age 40–44 years. The grave contained also a man a woman and 2 fetuses. There were a malachite necklace, a basket and ceramic objects. The right sided facial bones were destroyed by the infection. The mandible was not affected.

Lesions such as those in Fig. 2 are not specific for Leishmaniasis. Such lesions are also seen in cancer, leprosy, trepanosomiasis, tuberculosis and other chronic infections [20]. Modern molecular techniques applied to small boney slivers removed from the margin of the lesions, however, allowed us to establish the diagnosis of Leishmaniasis in the skulls.

### Molecular analysis

**Figure 3:**
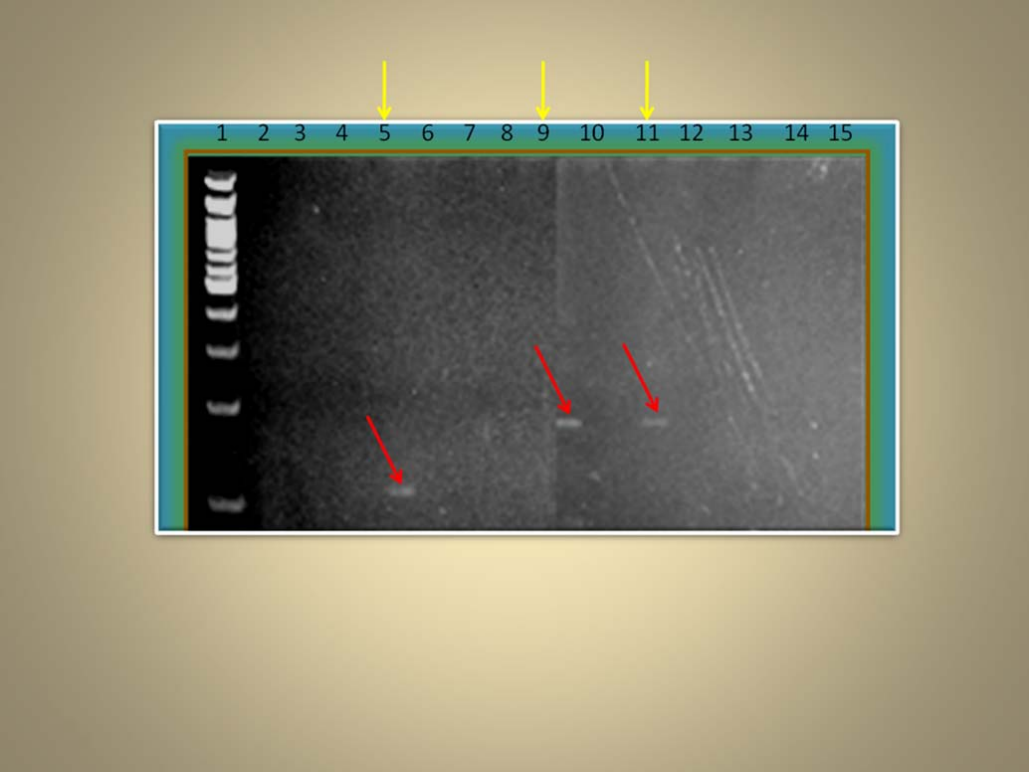
Polyacrylamide gel electrophoresis of samples 1 to 3 using primer sets LD1F/LD1R and LD3F/LD3R. Lane 1 contains a 100 bp molecular marker; lanes 2 to 6 and 9 to 13 contain two extracts of samples 1 and 2, and one extract of sample 3 respectively; lanes 7 and 14 contain an extract negative and lanes 8 and 15 contain a PCR negative. Red arrows arrows to indicate the position of the amplified DNA on the gel images.

**Figure 4:**
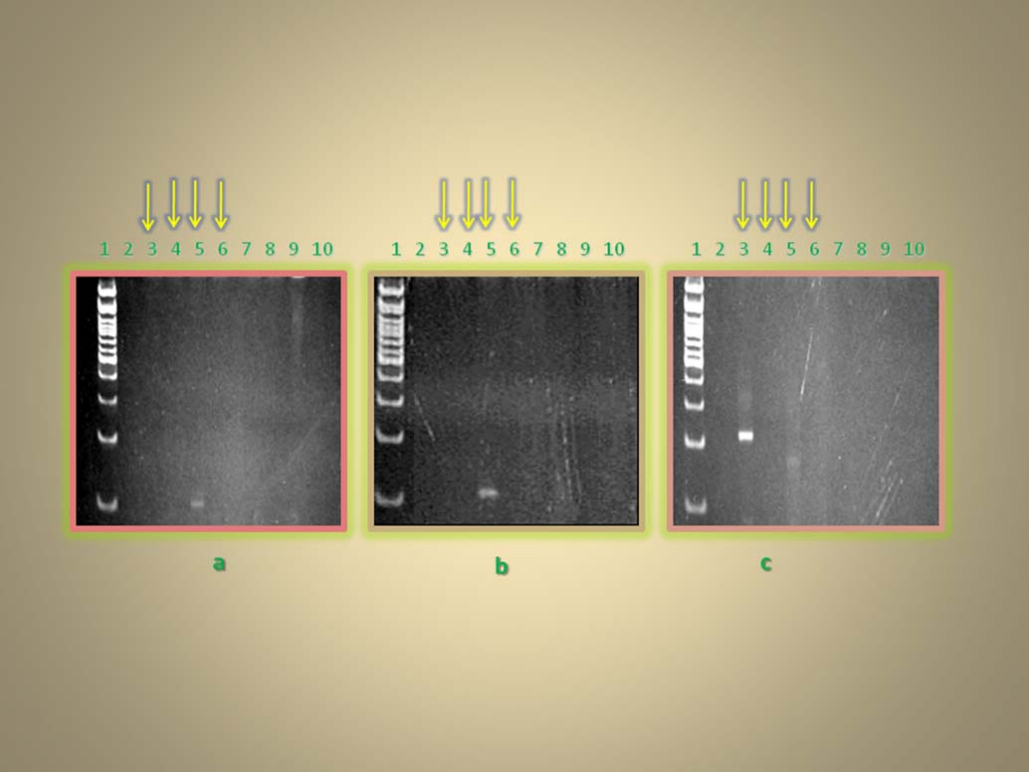
Polyacrylamide gel electrophoresis of samples 1 to 4 using primer sets LD1F/LD1R (a), LD2F/LD2R (b) and LD4F/LD4R (c). Lane 1 contains 100 bp molecular marker; lane 2 is blank; lanes 3 to 6 represents samples 1, 2, 3, 4 respectively; lanes 8, 9, 10 contain the extract negative, purification negative and PCR negative respectively.

We began by amplifying DNA from tissue sections of samples 1 to 3, using four different primer sets, each specific for a unique species-specific gene. The analysis produced one 118 bp fragment of the IMP dehydrogenase gene from sample 2 and one 184 bp fragment of the kinetoplast minicircle from samples 1 and 2 (Figure 3) indicating successful amplification. Next we examined bone fragments of all four samples with each primer set. Amplification resulted in a 118 bp fragment of the IMP dehydrogenase gene and 131 bp fragment of the amino acid permease AAP13LD gene from sample 3, and a 220 bp fragment of the adenylate kinase gene from sample 1 (Figure 4). Cloning and sequencing of these products confirmed the presence of Leishmania DNA. However the sequence differs from *L. donovani*, as expected if these were American forms of Leishmania (see supporting on line information; text S3).

## Discussion

We identified disfiguring facial boney lesions, characteristic of Leishmaniasis, in 4 women who died in a high altitude desert community where Leishmaniasis is not found. To confirm the etiology of this disfiguring disease we used molecular methods which identified the causative organism in 3. The fourth was assigned to the same causative agent by inference.

The unprecedented discovery of ancient facial lesions in 4 female skulls, shown to be caused by Leishmaniasis in a non-endemic area requires socio-archaeological explanations.

The aridity and altitude of San Pedro de Atacama makes it unlikely that the disease ever existed in the area. The affected individuals must have been immigrants to San Pedro de Atacama from an endemic area. They were integrated into the local community where they spent the rest of their lives and died. How and from where could these individuals have arrived in San Pedro de Atacama?

We propose that the infection occurred on the Eastern slopes of the Andes, where Leishmaniasis is endemic; a distance of some 400 km from San Pedro de Atacama. Thus the infected individuals could have been natives of San Pedro de Atacama who traveled to the endemic zone, were infected with Leishmaniasis and then returned to their native high altitude desert. Alternatively, they could have been natives of the distant endemic area who migrated to San Pedro de Atacama after they became infected. The long incubation period between infection and the development of destructive facial and boney lesions–up to 20 years [21]—would have allowed plenty of time for infected travelers to complete their journey before they became debilitated.

Noteworthy is that all 4 skulls were female. We favor the notion that these women were immigrants to the Atacama Desert and arrived there while still young, before they developed the facial disfigurement. The known exchanges of goods between Atacama communities and the Eastern Andean slopes may also have facilitated social interactions, such as marriage, between these widely separated communities. Although the considerable distance of the endemic area from San Pedro de Atacama could have discouraged travel, the populations mingled at “nodal points” along the way [3], where Atacameños traded for the hallucinogen (cebil) they prized. Young men may have found wives at these nodal points, including young women who had been infected with leishmaniasis in their homeland, but who did not yet show signs of the disease because of the long latency before the appearance of chronic facial ulcerations. Such a scenario could also apply to men. But the lack of evidence of male infection in San Pedro de Atacama suggests that such alliances were “patrilocal” –that is only women followed men to their native communities.

Our conclusions rest not only on archaeological evidence but also on an improved method of detection for Leishmaniasis DNA (see supporting on-line information; text S2, S3) with increased sensitivity and specificity for this parasite. The assay enabled us to amplify fragmented DNA from a variety of ancient tissues, and the results were highly specific: Only DNA of our target species was amplified.

*L. donovani* DNA has been extracted from Egyptian and Nubian mummies dated to 4000 years ago [22]. This organism caused visceral Leishmaniasis, the uniformly fatal form of the disease.

By contrast, the muco-cutaneous leishmaniasis we have detected in ancient South American DNA is a chronic disfiguring form of the disease that allowed prolonged survival and normal social interactions during the lengthy incubation period, before permanent disfigurement. The Leishmaniasis DNA data in publicly accessible databases comes mainly from two species, *Leishmania major* and *Leishmania donovani*.

Unfortunately, Only one gene (LDR3) sequenced to usable quality in our study. Each of the other sequences was of poor quality and therefore was not included in the final analysis. Two of the four archeological samples produced readable sequences. Because of the number of polymorphisms in the sequence (see supporting on line information; text S3) we cannot be sure that these sequences were those of L. Donovani. Also, more recent information, implies that L. infantum may have been sequenced from the ancient boney material (see supplementary on line information text S3).

The correct species of the ancient parasite in the desert of San Pedro de Atacama, a non-endemic area, has yet to be determined. The sequence we generated does not exactly match that of *L. donovani*. The differences could be used to identify the species.

Strict contamination controls were employed in our analysis (see supporting on line information; text S2). No Leishmaniasis DNA had ever before been analyzed in this laboratory. The PCR products were cloned and sequenced and one primer set was replicated independently in a different laboratory.

The sensitivity, specificity, and the rapid and reproducible nature of our method have proven highly effective in analyzing this archaeological material. The identification of this disease in a non-endemic area, at high altitude, in South America and a comparison of the ancient Leishmaia DNA to that of the modern day species will provide useful information about the evolution of Leishmaniasis, its pathogenesis, and its spread throughout these regions.

The present globalization, characterized by cross–border movement of people, commodities, vectors, diseases, food and commerce, has extraordinary potential to influence the spread and emergence of infectious diseases. We record here that such factors were at work millennia ago; they must have shaped demographic trends and the spread of infectious diseases throughout pre-history. These factors have implications for prevention and control of emerging and re-emerging infections such as Leishmaniasis [23].

## Materials and Methods

Coyo Oriente is a prehistoric cemetery excavated by Paige [24]. Two hundred and ninety three individuals from 160 tombs were examined. Only well preserved skulls were entered into the museum's collection; which now houses 255 skulls from this cemetery (see supporting on line information Table S1.). In addition to pottery, weavings, caps and other textiles, 55 tablets used for the inhalation of hallucinogens and numerous baskets embroidered with colorful wools, also commonly found in the Argentine northwest [25], were exhumed and are preserved in the museum.

### Primers

Four genes within the Leishmania genome were chosen as targets to design four sets of oligonucleotide primers on the basis of literature search, stability and function within the genome using the sequence of *Leishmania donovani* as templates (see supporting on line information Table S2). Initial design began with locating a suitable sequence of bases within an 18 to 25 base-length and coupling with a second sequence to produce an amplicon size of no greater than 300 base pairs (bp). A standard nucleotide-nucleotide BLAST (blastn)[26] search was performed on each individual primer to verify the species-specific origin of the sequence in question, followed by the determination of melting temperature using QIAGEN's Oligos Toolkit (http://oligos.quiagen.com/oligos/toolkit.php).

Amplicon analysis continued with a computer simulated PCR program (Amplify version 1.2 for Mac software) to determine the primability and stability of match, and to visualize the sensitivity of each amplicon. (see supporting on line information; text S2, S3).

## Supporting Information

Text S1 (0.03 MB DOC)

Text S2 (0.03 MB DOC)

Text S3 (0.05 MB DOC)

Table S1 The skulls kept in the Instituto Investigaciones Arqueológicas y Museo, Universidad Católica del Norte, San Pedro de Atacama, Chile.
(0.04 MB DOC)

Table S2 Leishmania donovani. Primer list.
(0.03 MB DOC)
