# Supplementary material for: Ancient Leishmaniasis in a Highland Desert of Northern Chile
Source: PLoS One. 2009 Sep 10;4(9):e6983. doi: 10.1371/journal.pone.0006983 (PMC2735183; doi:10.1371/journal.pone.0006983)
Supplement: Text S1 — (0.03 MB DOC) [file pone.0006983.s001.doc]

**Supporting information text S1**

SEX ESTIMATION

The skull is the second best area of the skeleton (after pelvis) to use for determining sex. Estimation of sex is based on generalization that the male is more robust, rugged, and muscle marked than female.

Absolute differences seldom exist, and many intermediate forms are found, but distinguishing characteristics are as follows:

**A- Face**

1- Sopraorbital ridges are more prominent in males than in females

2-Upper edges of the eye orbits are sharp in females, blunt in males; orbits are round in females, squared in males. Glabella is highly pronounced in males.

3- The palate is larger in males

4- Teeth are larger in males

**B- Mandible**

1- The chin is more squared in males and rounded with a point in midline in females

2-Teeth are larger in males

**C- Vault**

1-The female skull is smaller, smoother and generally more gracile. The female skull retains the child characteristics of frontal and parietal bossing into adulthood.

2- Muscle ridges, especially in the occipital bone (nuchal crests) are larger in males. Inion is highly pronounced in males.

3- The posterior end of the zygomatic process extends as a crest farther in males, often much past the external auditory meatus.

4- Mastoid processes are larger in males, thinner in females

5- Frontal sinuses are larger in males
