# Supplementary material for: Ancient Leishmaniasis in a Highland Desert of Northern Chile
Source: PLoS One. 2009 Sep 10;4(9):e6983. doi: 10.1371/journal.pone.0006983 (PMC2735183; doi:10.1371/journal.pone.0006983)
Supplement: Text S2 — (0.03 MB DOC) [file pone.0006983.s002.doc]

**Supporting on line information: text S2.**

***PCR Optimization.*** (Excerpted from thesis by Iachetta, Lucia. (2006). Molecular Detection and Identification of Parasites Involved in Human Disease. ***Unpublished*** Thesis. Lakehead University. Thunder Bay, ON Canada.)

To facilitate primer optimization, a positive control of *Leishmania donovani* DNA was diluted into 1/100, 1/1 000 and 1/10 000 aliquots in order to test primer sensitivity and specificity. A 25.0μl reaction volume for PCR containing 1x PCR buffer, 1mM magnesium chloride (MgCl_2_), 0.2mM of each dNTP, 0.2μM of each forward and reverse primer, 0.02 units of *Taq* Polymerase and 10.0μl of DNA template. The reactions were carried with the following program: initial denaturation of two minutes at 94^o^C followed by 45 cycles of denaturation, primer annealing and primer extension at 94^o^C for one minute, 60^o^C for 1 minute and 72^o^C for two minutes respectively. Following amplification, PCR products were analyzed by electrophoresis on 6% polyacrylamide gel using a 100bp molecular marker (2.0μl) (Fermentes) for size approximation, loaded with 5.0μl of PCR product and 3.0μl of 6X loading dye [2.5% xylene cyanol (12.0μl), 2.5% bromophenol blue (12.0μl), 35% Ficoll (432.0μl) and 544.0μl of ddH_2_O and a 15 to 20 minute staining period in ethidium bromide (EtBr) solution (1/20 dilution using 5.0μl of EtBr stock solution by STRATAGENE ®).

***Extraction/Purification*** Sample preparation began with steps to eliminate contaminating modern DNA. These included UV irradiation, surface removal and pre-wash treatment steps. The samples were then prepared by grinding of the muscle tissue attached to the skull and the bone flakes from the skull, nasal and orbital regions, with a pestle in the microcentrifuge tube to be used for DNA extraction. DNA was extracted from this tissue by a modified silica-guanidinium thiocyanate (GuSCN) procedure [1]. P-30 spin columns were used to further purify the extract. DNA extraction of the bone flakes employed the phenol:chlorofom separation method followed by ethanol precipitation. Application of P-30 spin columns proceeded by 10% Chelex incubation added to the purification of the extract.

***PCR*** A 25.0μl reaction volume for PCR containing 1x PCR buffer, 2mM magnesium chloride (MgCl_2_), 0.2mM of each dNTP, 0.2μM of each forward and reverse primer, 0.06 units of Taq Polymerase and 15.0μl of DNA template. The reactions were conducted in 50 cycles with the following program: initial denaturation of two minutes at 94^o^C followed by 60 cycles of denaturation, primer annealing and primer extension at 94^o^C for one minute, 60^o^C for 30 seconds and 72^o^C for two minutes respectively. PCR products (5.0µl) were added to 3.0µl of 6X loading dye (produced in laboratory): 12.0µl of 2.5% xylene cyanol, 12.0µl 2.5% bromophenol blue, 432.0µl of 35% Ficoll and 544.0µl of ddH20) and electrophoresed in a 6.0% (w/v) polyacrylamide gel in 1 X TBE at 120 volts for 45 minutes. Amplified DNA was visualized by ethidium bromide staining exposed to ultraviolet light and photographed.

***Cloning*** PCR products were cloned using T4 DNA ligase to insert the DNA fragment of interest into a pure linear pUC19 vector. The ligated vector was introduced into competent *E. coli* JM109 cells and grown overnight on LB Agar containing X-Gal and IPTG to identify successful pUC19 insertion. The resulting glossy, white colonies were plucked and cultured overnight in LB broth. The isolation and purification of the resulting DNA sequence was accomplished with the QIAprep plasmid purification system and amplified via PCR with sequence specific primers. Sequencing of PCR products occurred in a reaction of 40 cycles of 96oC for 30 seconds, 50oC for 15 seconds and 72oC for four minutes employing the ABI Prism® 3100 Genetic Analysis instrument.

***General Laboratory Procedures*** To guard against contamination of the sample with extraneous DNA, several precautions were taken: (i) a full-body, hooded Tyvek suit was worn throughout the entire extraction procedure, as well as a face mask, goggles and double gloves that were frequently changed when moving stations; (ii) the sampling and extractions were carried out in laminar flow hoods located within a room devoid of any history of work with modern leishmaniasis; (iii) PCR preparation and analysis was performed in three separate areas, one area for PCR reagent set-up (hood), one area for PCR itself, and one area for PCR analysis; (iv) sterile disposable laboratory instruments were utilized whenever possible, as well as positive displacement pipettes and barrier-tipped pipette tips, with each area containing its own set of equipment that were not interchanged; (v) all areas prior to and following use were thoroughly cleaned using 10% sodium hypercholorite, ddH_2_O, 70% ethanol, and hoods were UV irradiated for at least one hour; (vi) extraction and reagent blanks (negative controls) were simultaneously run alongside with the detection PCR.

***References.***

1. Boom R, Sol CJ, Salimans MM, Jansen CL, Wertheim-van Dillen PM, et al. (1990) Rapid and Simple Method for Purification of Nucleic Acids Journal of Clinincal Microbiology 28(3): 495-503
