# Supplementary material for: Ancient Leishmaniasis in a Highland Desert of Northern Chile
Source: PLoS One. 2009 Sep 10;4(9):e6983. doi: 10.1371/journal.pone.0006983 (PMC2735183; doi:10.1371/journal.pone.0006983)
Supplement: Text S3 — (0.05 MB DOC) [file pone.0006983.s003.doc]

An important issue is the primer specificity. To this end we can say, with assurance, that at **the time of the design of our study**, using NCBI nucleotide blast, that the primers were **100% matched to the intended species (*Leishmania sp)* and corresponding gene and to no other species or gene.**

However these computer based tests are only as reliable as the DNA sequence data within them. The primers were, therefore, experimentally tested by us on *Mycobacterium tuberculosis*, *Treponema pallidum* and *Mycobacterium leprae* and found to be negative.

Also the LD3R primer had previously been published^1^ in this study it was checked on other pathogens as well and also found to be negative.

[1] Salotra P G Sreenivas GP Pogue N Lee HL Nakhasi V et al. (2001). Development of a Species-Specific PCR Assay for Detection of Leishmania donovani in Clinical Samples from Patients with Kala-Azar and Post-Kala-Azar Dermal Leishmaniasis. J. Clin Microbiol 39: 849-854.

After our study was completed additional work came to light:

GENBANK ACCESSION #: AM502252.1 - L. infantum

Peacock et al (2007). Nature Genetics 39(7):839-847

GENBANK ACCESSION #: DQ452734.1 - L. infantum

Ravel et al (2006). International Journal for Parasitology 36(13):1383-1388

GENBANK ACCESSION #: XM_001686050.1 - L. major

Iven et al (2005). Science 309(5733):436-442

GENBANK ACCESSION #: XM_001468363.1

Peacock et al (2007).  Unpublished L. infantum

Although the above primers hit the targets of the genes shown in each of the citations, in 2 of the references (Ravel et al 2006 and Iven et al 2005, see above), only one primer set hit those targets.  Theoretically, therefore, amplification of that gene target should not have occurred. This excludes L. major from the list.  It is only when the gene targets both primers (the forward and reverse primer) that amplification of DNA should occur. This includes the references by Peacock et al from 2007 which are both representative of **L. infantum**.

In the light of the above, more recent information, it is also possible that we amplified L. infantum

**Below are details of the primer sets and sequences:**

**MODERN LEISHMANIA DNA GEL IMAGES**

M 2 4 6 8


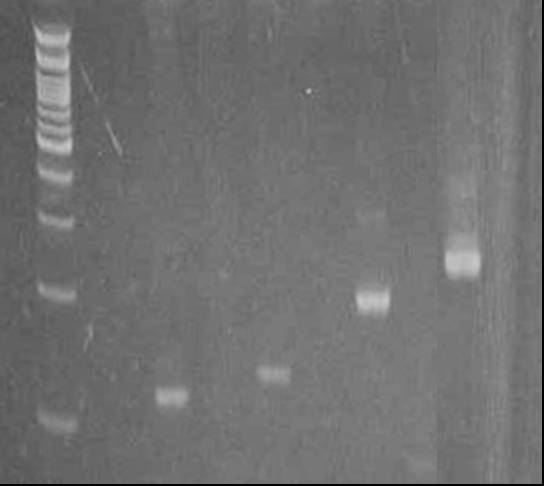


**Figure 1. 6% PAGE image of modern Leishmania donovani DNA amplification with the 4 primer sets.**

Image schematics: M represents 100bp molecular marker; lanes 2, 4, 6, 8 represent reaction involving primer sets LD1F/LD1R, LD2F/LD2R, LD3F/LD3R and LD4F/LD4R respectively; lanes 3, 5, 7, 9 represent corresponding PCR negatives.

**MODERN LEISHMANIA SEQUENCES**

**LD1 GENE** AAGACCATCA AGGATGGCTG CACCGCCGAG GAGTTGTTCC GGGGCGATGG ACTGACGTA.

LD1F AAGACCATCA AGGATGGCTG CACCGCCGAG GAGCTGTTCC GGGGCGATGG ACTGACGTA.

**LD2 GENE** GAAGTGGTAC GATGGATGCT AATCATTTAT AACACGGGAT CTGCTATTGG CTACCTCGTC

LD2F GAAGTGGTAC GATGGATGCT AATCATTTAT AACACGGGAT CTGCTATTGG CTACCTCGTC

**LD2 GENE** GTCTT..... .......... .......... .......... .......... ..........

LD2F GTCTT..... .......... .......... .......... .......... ..........

**LD3 GENE** ACTATATTAT CGGTAGTATA ATATCATAAG TATACGGTAT AGATATATGT TAATTGTAGT

LD3F ACTATATTAT CGGTAGTATA ATATCATAAG TATACGGTAT AGATATATGT TAATTGTAGT

**LD3 GENE** ATATTGTAGA TCTATGTTAC AGTGTATAGT CTATGAACTT ACTAGATATA ATTTGTATTT

LD3F ATATTGTAGA TCTATGTTAC AGTGTATAGT CTATGAACTT GCTAGATATA ATTTGTATTT

**LD3 GENE** GATGCTATAG TGCTACTGAT AGAGTGTACC TA........ .......... ..........

LD3F GATGTTATAG TGCTACTGAT AGAGTGTACC TA........ .......... ..........

**LD4 GENE** CGTCAATGGC TTCGTGCTGG ACGGTTTTCC GCGCACCCGC AAGCAGTCGA GGATGATGCA

LD4F CGTCAATGGC TTCNTGCTGG ACGGTTTTCC GCGCACCCGC AAGCAGTCGA GGATGATGCA

**LD4 GENE** AGATTTGGAG AACGTGAAAG TCGACATTGT GGTCGAGTTG GAGATTTCGG ACAAAGAGCT

LD4F AGATTTGGAG AACGTGAAAG TCGACATTGT GGTCGAGTTG GAGATTTCGA ACAATGAGCT

**LD4 GENE** ACAGACTCGC TTC....... .......... .......... .......... ..........

LD4F ACAGACTCGC TTC....... .......... .......... .......... ..........

Figure 2. Modern DNA sequences which correspond to the gel image (above).

Highlighted (in yellow) the published gene from which each primer set was obtained for comparison with experimental sequences, amplified from the modern *Leishmania donovani* DNA (Figure 1).

Sequence alignment of the four genes analyzed in *Leishmania donovani*. Alignment illustrates the published gene fragment sequence followed by the experimentally attained sequence of the corresponding primer. Sequence polymorphisms highlighted in red.

**ANCIENT LEISHMANIA SEQUENCES**

**LD3 GENE** GGAGTAGCCT CAGGACTTTA GGCGGGAGAT ACTATATTAT CGGTAGTATA ATATCATAAG

SAMPLE 1 F ---------- ---------- ---------- ---------- ---------- ----------

SAMPLE 2 F ---------- ---------- ---------- ---------- ---------- ----------

LD3F ---------- ---------- ---------- ACTATATTAT CGGTAGTATA ATATCATAAG

**LD3 GENE** TATACGGTAT AGATATATGT TAATTGTAGT ATATTGTAGA TCTATGTTAC AGTGTATAGT

SAMPLE 1 F ---------- ---------- -----GTAGT ATATTGTAGA TCTATGTTAC AGTGTATAGT

SAMPLE 2 F ---------- ---------- ---------- ---------- ---------- ---GTATAGT

LD3F TATACGGTAT AGATATATGT TAATTGTAGT ATATTGTAGA TCTATGTTAC AGTGTATAGT

**LD3 GENE** CTATGAACTT ACTAGATATA ATTTG-TAT- --TTGATGCT ATAGTGC-T- ACTGATAGAG

SAMPLE 1 F CTATGGACTT GCTAGATATA ATTTG-TAT- --TTGATGTT AGAGTGC-T- ACTGATAGAG

SAMPLE 2 F CTATGAACTT GCTAGATATA ATTTGGTAT- --TTGATGTT ATAGTGC-T- ACTGATAGAG

LD3F CTATGAACTT GCTAGATATA ATTTG-TAT- --TTGATGTT ATAGTGC-T- ACTGATAGAG

**LD3 GENE** TG-TACCTAT CACTAGT... .......... .......... .......... ..........

SAMPLE 1 F TG-------- -------... .......... .......... .......... ..........

SAMPLE 2 F TG-TACCTA- -------... .......... .......... .......... ..........

LD3F TG-TACCTA- -------... .......... .......... .......... ..........

Figure3. The amplified sequences from the archaeological material and sequence alignments of the archaeological samples, the published genes for *Leishmania donovani* and the experimentally attained sequence.

Only one gene (LDR3) sequenced to usable quality.  Each of the other sequences was poor quality and therefore was not included in the analysis.  The results show that 2 of the 4 samples (samples 1 & 2) produced readable sequences. Because of the number of polymorphisms in the sequence we did not state with assurance that these sequences were those of *L. donovani*. Compare ancient sequences to those of the published sequence (highlighted in yellow) and modern sequence from Figure 2 (highlighted in pink).
