# Supplementary material for: Ancient Leishmaniasis in a Highland Desert of Northern Chile
Source: PLoS One. 2009 Sep 10;4(9):e6983. doi: 10.1371/journal.pone.0006983 (PMC2735183; doi:10.1371/journal.pone.0006983)
Supplement: Table S1 — The skulls kept in the Instituto Investigaciones Arqueológicas y Museo, Universidad Católica del Norte, San Pedro de Atacama, Chile. (0.04 MB DOC) [file pone.0006983.s004.doc]

|  | Sex | | |  |  |
| --- | --- | --- | --- | --- | --- |
| Age | Female | Male | subadults | Indeterminate | Total |
| 2 | - | - | 2 | - | 2 |
| 3 | - | - | 2 | - | 2 |
| 4 | - | - | 1 | - | 1 |
| 5 | - | - | 1 | - | 1 |
| 6 | - | - | 2 | - | 2 |
| 8 | - | - | 2 | - | 2 |
| 10 | - | - | 1 | - | 1 |
| 11 | - | - | 1 | - | 1 |
| 12 | - | - | 2 | - | 2 |
| 15 | - | 1 | - | - | 1 |
| 15-19 | 3 | - | - | - | 3 |
| 25-29 | 1 | - | - | - | 1 |
| 30-34 | 17 | 13 | - | - | 30 |
| 35-39 | 39 | 22 | - | - | 61 |
| 40-44 | 22 | 27 | - | - | 49 |
| 45-49 | 25 | 24 | - | - | 49 |
| 50-54 | 6 | 7 | - | - | 13 |
| 55-59 | 1 | 4 | - | - | 5 |
| Adult | 5 | 4 | - | - | 9 |
| Mature adult | 7 | 5 | - | - | 12 |
| Senile | 2 | - | - | - | 2 |
| Indeterminate | 1 | - | 1 | 4 | 6 |
| Total | 129 | 107 | 15 | 4 | 255 |

**Table 1:  The skulls kept in the Instituto Investigaciones Arqueológicas y Museo, Universidad Católica del Norte, San Pedro de Atacama, Chile.**

The archeological cemetery Coyo Oriente, structure of the sample used in this study, by sex and age.
