# Supplementary material for: Ancient Leishmaniasis in a Highland Desert of Northern Chile
Source: PLoS One. 2009 Sep 10;4(9):e6983. doi: 10.1371/journal.pone.0006983 (PMC2735183; doi:10.1371/journal.pone.0006983)
Supplement: Table S2 — Leishmania donovani. Primer list. (0.03 MB DOC) [file pone.0006983.s005.doc]

| **Name** | **Sequence 5'-3'** | **Location in *Leishmania donovani*** | **Base Pair (bp)** | **Temperature (^o^C)** | **Source** |
| --- | --- | --- | --- | --- | --- |
| LD1F | TGC GTG CGT GTG TGT GAC ATC | IMP dehydrogenase gene | 118 | 65.32857 | Iachetta (2006) * |
| LD1R | TAC GTC AGT CCA TCG CCC CG | IMP dehydrogenase gene |  | 66.55 | “ |
| LD2F | AGA CAA GCT GGG TCG TAA CTC | amino acid permease AAP13LD gene | 131 | 63.37619 | “ |
| LD2R | AAG ACG ACG AGG TAG CCA ATA G | amino acid permease AAP13LD gene |  | 63.4 | “ |
| LD3F | GGA GTA GCC TCA GGA CTT TAG | kinetoplast minicircle | 184 | 63.7619 | “ |
| LD3R | TAG GTA CAC TCT ATC AGT AGC AC | kinetoplast minicircle |  | 61.72609 | (Salot*ra et a*l., 2001)# |
| LD4F | GCT GGA AGT GAA GGT TGT CTG C | adenylate kinase | 220 | 65.26364 | Iachetta (2006) |
| LD4R | GAT AGA TGC GTC CCG ACT TGG | adenylate kinase |  | 65.328575 | ” |

**Table 2. Leishmania donovani.** Primer list.

***** Iachetta Lucia. (2006). Molecular Detection and Identification of Parasites Involved in Human Disease. Unpublished Thesis. Lakehead University. Thunder Bay, ON Canada.

**#** Salotra P G Sreenivas GP Pogue N Lee HL Nakhasi V et al. (2001). Development of a Species-Specific PCR Assay for Detection of Leishmania donovani in Clinical Samples from Patients with Kala-Azar and Post-Kala-Azar Dermal Leishmaniasis. J. Clin Microbiol 39: 849-854.
